# Cerebrospinal Fluid Galectin-1 Levels Discriminate Patients with Parkinsonism from Controls

**DOI:** 10.1007/s12035-018-1426-9

**Published:** 2018-11-21

**Authors:** Tainá M. Marques, Anouke van Rumund, Ilona B. Bruinsma, Hans J. C. T. Wessels, Jolein Gloerich, Rianne A. J. Esselink, Bastiaan R. Bloem, H. Bea Kuiperij, Marcel M. Verbeek

**Affiliations:** 1 Department of Neurology, Donders Institute for Brain, Cognition and Behaviour, Radboud University Medical Center, Nijmegen, The Netherlands; 2 Department of Laboratory Medicine, Radboud University Medical Center, Nijmegen, The Netherlands; 3 Parkinson Center Nijmegen, Nijmegen, The Netherlands

**Keywords:** Parkinson’s disease, Biomarkers, Galectin-1, Cerebrospinal fluid, Validation

## Abstract

Parkinson’s disease (PD) is the second most common neurodegenerative disorder in elderly people. Currently, the diagnosis of PD is based on neurological examination, neuroimaging, and the response to dopaminergic medication. The diagnosis can be challenging, especially at early disease stages, when the symptoms of patients with atypical parkinsonism (APD) may strongly overlap. Therefore, reliable biomarkers that are able to identify patients with PD are much needed. Here, we aimed to identify and validate new biomarkers for PD in cerebrospinal fluid (CSF). We performed a profiling experiment using mass spectrometry (MS) of CSF from ten PD patients and ten matched non-neurological controls. We selected one protein, galectin-1 (Gal-1), which was differentially expressed in PD vs. controls, and quantified its concentrations in CSF by enzyme-linked immunosorbent assay (ELISA) in three new cohorts of 37 PD patients, 21 APD patients, and 44 controls. CSF levels of Gal-1 were lower in PD in both the discovery and validation experiments and discriminated PD from controls with moderate–high accuracy levels (ELISA: area under the curve = 0.7). Similar levels of Gal-1 were found in PD and APD. Gal-1 levels were correlated to age in all groups and correlated in the PD patients to CSF levels of total tau, phosphorylated tau, neurofilament light chain (NFL), and the mini-mental state examination (MMSE) score. We conclude that MS profiling of proteins may be a useful tool to identify novel biomarkers of neurological diseases and that CSF Gal-1 levels may discriminate PD from non-neurological controls.

**Electronic supplementary material:**

The online version of this article (10.1007/s12035-018-1426-9) contains supplementary material, which is available to authorized users.

## Introduction

Parkinson’s disease (PD) is one of the α-synucleinopathies and the most prevalent neurodegenerative movement disorder. Accumulation of α-synuclein (α-syn) in dopaminergic neurons in PD leads to neuronal death causing motor and non-motor dysfunction. PD motor symptoms include bradykinesia, muscular rigidity, impaired balance, and resting tremor. Non-motor symptoms include sleep disorders, olfactory dysfunction, autonomic dysfunction, and cognitive impairment [1, 2].

PD diagnosis is based on neurological evaluations, neuroimaging, and the response to dopaminergic medication following the current international clinical criteria [3, 4]. However, especially at early stages of diseases, the symptoms and signs of patients with atypical parkinsonisms (APD), such as multiple system atrophy (MSA) and progressive supranuclear palsy (PSP), may strongly overlap with PD, leading to misdiagnosis and incorrect choice of treatment. Therefore, reliable biomarkers that could identify PD are dearly needed.

Cerebrospinal fluid (CSF) is the body fluid that is closest to the brain, and its composition may reflect the (pathological) processes in the brain. Several proteins that were already known to be involved in PD have been investigated in CSF as potential biomarkers for diagnosis, disease progression, or cognitive decline, such as α-syn, neurofilament light chain (NFL), DJ-1, tau, and amyloid β42 (reviewed in [5]). A consistent moderate reduction in CSF α-syn levels has been described not only in PD but also in other α-synucleinopathies (reviewed in [6]). Although many studies have been identified potential biomarkers for PD, none of them has yet reached clinical practice.

We aimed to identify new CSF biomarkers that have the potential to discriminate PD from controls. For this purpose, we selected PD patients from a unique cohort of patients with uncertain diagnosis of parkinsonism at presentation, which is very representative of the daily situations when clinicians are confronted with patients with the suspicion of a movement disorder at the first visit to a specialist. We performed a profiling experiment by mass spectrometry in CSF of ten PD patients and ten non-neurological controls. We selected the protein galectin-1 (Gal-1), which was differentially expressed in PD vs. controls, for further validation studies, using an enzyme-linked immunosorbent assay (ELISA).

## Methods

### Patients and CSF

We have selected CSF samples from ten PD and ten non-neurological control patients for the discovery experiment matched for sex and age. For the validation experiment, we have included CSF from 37 PD patients, 21 APD patients (MSA = 14; PSP = 7), and 44 non-neurological controls. Samples were matched for sex in all groups and for age in PD and controls, since age was higher in the APD group. We used separate cohorts of patients for the discovery and validation phases of the study, with the exception of only one PD CSF sample, which was used for both discovery and validation experiments. CSF samples for the discovery experiment were also selected based on low number of leukocytes (0–5 cells per μL) and erythrocytes (≤ 200 cells per μL). Demographic characteristics for both cohorts are shown in Table 1.

**Table 1:** Group characteristics

	Discovery			Validation			
	Control	PD	*p* value*	Control	PD	APD	*p* value*
Demographics							
*n*	10	10		44	37	21	
Age at inclusion (years)	59 ± 7	61 ± 8	*p* = 0.4	58 ± 10	57 ± 10	64 ± 7	*p = 0.02*
Sex (male/female)	6/4	7/3	*p* = 1.0	21/23	24/13	13/8	*p* = 0.26
Disease duration (months)	NA	37 ± 17		NA	36 ± 34	31 ± 23	*p* = 0.92
DM (no/yes)	NA	9/1		NA	31/6	14/7	
CSF parameters							
Gal-1^#^	5E+06 ± 4E+06	6E+05 ± 2E+06	*p = 0.02*	6961 ± 3475	6046 ± 2631	7133 ± 3795	*p* = 0.46
Total protein (mg/L)	468 ± 66	492 ± 54	*p* = 0.4	547 ± 644	530 ± 182	591 ± 273	*p < 0.001*
Gal-1/total protein (ng/mg)	NA	NA		17 ± 9	11 ± 5	14 ± 9	*p = 0.003*
α-Syn (μg/L)	NA	24 ± 9		NA	*n* = 37	*n* = 20	*p* = 0.60
					29 ± 12	30 ± 12	
NFL (ng/L)	NA	1415 ± 528		NA	*n* = 37	*n* = 20	*p < 0.0001*
					1122 ± 639	4511 ± 3633	
Total tau (ng/L)	NA	273 ± 150		NA	*n* = 37	*n* = 21	*p* = 0.55
					202 ± 71	260 ± 111	
Phosphorylated tau (ng/L)					*n* = 37	*n* = 21	*p* = 0.92
	NA	58 ± 27		NA	48 ± 16	49 ± 15	
Disease severity							
Baseline							
H&Y score	NA	*n* = 10		NA	*n* = 36	*n* = 21	*p < 0.0001*
		2 ± 0			2 ± 1	3 ± 1	
UPDRS score	NA	*n* = 10		NA	*n* = 35	*n* = 21	*p = 0.046*
		22 ± 6			26 ± 13	33 ± 13	
ICARS score	NA	*n* = 9		NA	*n* = 33	*n* = 16	*p < 0.001*
		2 ± 2			2 ± 3	10 ± 11	
MMSE score	NA	*n* = 10		NA	*n* = 37	*n* = 20	*p* = 0.10
		29 ± 1			28 ± 2	27 ± 3	
Follow-up							
H&Y score	NA	*n* = 9		NA	*n* = 35	*n* = 14	*p < 0.0001*
		2 ± 1			2 ± 1	4 ± 1	
UPDRS score	NA	*n* = 7		NA	*n* = 33	*n* = 11	*p = 0.049*
		28 ± 10			29 ± 13	37 ± 9	
ICARS score	NA	*n* = 7		NA	*n* = 30	*n* = 11	*p < 0.001*
		2 ± 1			3 ± 3	12 ± 10	
MMSE score	NA	*n* = 8		NA	*n* = 30	*n* = 11	*p* = 0.05
		29 ± 1			28 ± 3	26 ± 3	

Values are expressed as mean ± standard deviation

*n* number of samples, *DM* intake of dopaminergic medication at CSF collection, *CSF* cerebrospinal fluid, *Gal-1* galectin-1, *α-syn* α-synuclein, *PD* Parkinson’s disease, *APD* atypical parkinsonism, *NA* not applicable, *H&Y* Hoehn and Yahr score, *UPDRS* Unified Parkinson’s Disease Rating Scale, *ICARS* International Cooperative Ataxia Rating Scale, *MMSE* mini-mental state examination score

^#^Units: in discovery cohorts, arbitrary intensity; in validation cohorts, ng/L

*Parameters were analyzed with Kruskal–Wallis test when data of the three groups was available. For comparisons between two groups Student’s *t* test or Mann–Whitney *U* test, except for sex, which was analyzed using chi-squared test; In italic *p*-value below 0.05

PD and APD patients for the discovery and validation cohorts were selected from a longitudinal study performed at the Radboud University Medical Center, previously described in detail (Nijmegen, The Netherlands) [7]. These cohorts included patients who were referred to our tertiary center between January 2003 and December 2006 with an uncertain and yet undefined diagnosis of parkinsonism. At baseline, an extensive array of ancillary diagnostic tests was performed, among which a lumbar puncture to allow biomarker studies. These patients had been followed up for 3 years at each time a team of movement disorders specialists determined a final clinical diagnosis. The diagnosis of PD or APD was based on established criteria for PD [8], MSA [9], or PSP [10] at the time of 3-year follow-up and updated according to the most recent clinical criteria [3, 4, 11]. Clinical parameters were obtained from PD and APD patients both at baseline and after 3 years of follow-up, including disease severity and cognitive function, using the Hoehn and Yahr (H&Y) scores [12], Unified Parkinson’s Disease Rating Scale (UPDRS) [13], International Cooperative Ataxia Rating Scale (ICARS) [14], and mini-mental state examination (MMSE) [15]. The use of dopaminergic medication was registered at the time of lumbar puncture, in order to include as a possible confounding factor. CSF levels of α-syn, total tau, phosphorylated tau, and NFL concentrations in the PD patients were previously published by our group by using various ELISAs (see Table 1) [16–18]. Details of the methods for the quantification of these CSF parameters have been described in detail [16–18].

The non-neurological control group consisted of patients who were referred to our center with a suspicion of neurological disease, but after extensive neurological examination had no neurological disorder and were diagnosed with other disorders, such as non-neuronal sarcoidosis, diabetes, radiculopathy, or headache. Their CSF did not show any abnormality for the following parameters: cell count, glucose, total protein, lactate, hemoglobin, bilirubin, and oligoclonal IgG bands.

CSF samples of PD, APD, and non-neurological controls were collected in polypropylene tubes, centrifuged, aliquoted, and stored in polypropylene tubes at − 80 °C until experiments. All participants provided written informed consent, and the study was approved by the local institutional review board Arnhem-Nijmegen.

### Mass Spectrometry Profiling

Total protein concentration in CSF was determined using the 2D Quant kit (GE Healthcare Life Sciences, UK), according to the manufacture’s protocol, and 400 μg of total protein for each sample was used as input for the discovery experiment. CSF samples were applied to an affinity removal column for depletion of 14 most abundant proteins (MARS-14, Agilent Technologies, Santa Clara, CA, USA) to enrich low-abundant proteins.

Samples were diluted in 8 M urea to denature proteins prior to reduction in 10 mM dithiotreitol for 20 min at room temperature and alkylation with 50 mM chloroacetamide for 20 min at room temperature in the dark. Proteolytical digestion was performed by a first incubation with LysC protease for 3 h at 37 °C after which the sample was diluted 1:3 with 50 mM ammonium bicarbonate prior to overnight incubation with trypsin at 37 °C. Peptides were concentrated and desalted using C18 Omix tips (Agilent Technologies), eluted in 20 μL 80% acetonitrile, and dried using a SpeedVac centrifuge at 45 °C. Peptides were suspended in ammonium hydroxide buffer (pH 10) and subsequently fractionated using C18-reversed phase liquid chromatography (Waters Acquity UPLC; Waters Xbridge C18 3.5-μm particles, 1.0-mm ID × 100-mm length). Peptides were eluted from the column at 100 μL/min using a 15-min linear gradient of 5 to 45% acetonitrile adjusted to pH 10 with ammonium hydroxide. The 20 collected fractions were adjusted to pH 2.7 using formic acid prior to LC-MS/MS analysis.

Samples were analyzed by nanoflow liquid chromatography (Bruker Daltonics; nano-Advance) connected online to an ultra high-resolution quadrupole time-of-flight tandem mass spectrometer (Bruker Daltonics; maXis 4G ETD) via an axial desolvation vacuum-assisted electrospray ionization source (Bruker Daltonics; Captive Sprayer). Peptides were loaded onto the trapping column (Acclaim PepMap 100, 75 μm × 2 cm, nanoViper, 3-μm 100-Å C18 particles; Thermo Scientific) at 10 μL/min with 0.1% formic acid using two loop volumes of solvent (20 μL). Peptides were eluted from the analytical column (Acclaim PepMap RSLC, 75 μm × 15 cm, nanoViper, 2-μm 100-Å C18 particles; Thermo scientific) using a 20-min linear gradient of 5 to 35% acetonitrile in 0.1% formic acid at 600 nL/min. The mass spectrometer was operated in positive ion mode for data-dependent MS/MS acquisition. Each AutoMSn duty cycle consisted of one full MS spectrum (150–3700 *m*/*z*, 2-Hz spectrum acquisition rate) followed by six data-dependent MS/MS experiments acquired at intensity scaled spectral acquisition rates (3 Hz at 2000 counts, 16 Hz at 100,000 counts). Only precursor ions in the range of 400–1400 *m*/*z* with charge state of *z* = 2+ or higher were considered for collision-induced dissociation experiments with dynamic exclusion set to 2 min.

Raw MS data files were subsequently analyzed by MaxQuant software version 1.5 [19] using the predefined Qq-ToF parameter settings against the RefSeq (release 55) human protein sequence database with added contaminant protein sequences. Cysteine carbamidomethylation was specified as fixed modification whereas protein N-terminal acetylation, methionine oxidation, and deamidation of glutamine and/or asparagine as variable modifications. Label-free quantitation was performed using match between runs and re-quantify options using at least two razor and unique peptides. False discovery rate tolerances were set to 0.01 at both the peptide and protein level. Putative protein biomarkers were considered for subsequent validation based on *p* values calculated using the Mann–Whitney *U* test (*p* < 0.05) and should be quantified in at least 50% of the samples of any group.

### Gal-1 ELISA

Gal-1 was quantified in CSF by using a commercial ELISA (Human Galectin-1 PicoKine™ ELISA Kit; Boster Biological Technology, Pleasanton, CA, USA, Catalog #EK0762), according to the company’s protocol, with the exception of prolonged incubation times, as suggested by the company. Biotinylated anti-human galectin-1 antibody was incubated for 90 min, avidin–biotin–peroxidase complex (ABC) for 50 min, and color-developing agent for 30 min. CSF was diluted three times, and all samples were analyzed in duplicate.

We performed a partial validation of the ELISA according to previous recommendations [20, 21]. The detection limit was determined by measuring 18 blanks within one plate followed by calculation of the concentration that corresponds to the mean of all blanks plus three times the standard deviation.

The recovery was evaluated by spiking two different concentrations (6.7 ng/mL and 4.5 ng/mL) of Gal-1 recombinant protein in three times diluted CSF samples or in sample buffer. The percentage of spiked recombinant protein that was recovered from CSF was calculated. Values between 80% and 120% were considered satisfactory.

Precision was determined based on (1) intra-assay variation by measuring five diluted CSF samples in four replicates within one plate, and (2) inter-assay variation by measuring three diluted CSF samples in duplicate at identical positions in five different plates on five different days. The mean coefficient of variation (CV) was calculated; a CV below 20% was considered satisfactory.

CSF Gal-1 concentrations in the validation cohort were normalized by CSF total protein concentration to correct for a small significant difference of total protein concentration between PD (mean 530 ± 182 mg/L), APD (mean 591 ± 273 mg/L), and controls (mean 547 ± 652 mg/L, *p* = 0.004). Total protein concentration in CSF was measured by turbidimetric benzethonium chloride method using a Cobas 8000 instrument (Roche Diagnostics, Switzerland) for automated measurement.

### Data Analysis

Data analysis was performed using IBM SPSS Statistics 22 (Armonk, NY, USA) and GraphPad Prism 5 (La Jolla, CA, USA). Differences between groups were determined by ANOVA followed by Bonferroni post hoc test for normally distributed data and Kruskal–Wallis test followed by Dunn’s post hoc test for not normally distributed data. Mann–Whitney *U* test (in case of non-parametric data distribution) or Student’s *t* test (for parametric data) was used when data of only two groups were available. Analysis of covariance was performed with age and intake of dopaminergic medication as confounding factors. Correlations and partial correlations, with age and total protein concentration as covariates, were investigated by Spearman’s test. Receiver operating characteristic (ROC) curve was constructed to determine the diagnostic accuracy by the area under the curve (AUC) and the Youden index for the optimal cutoff values of sensitivity and specificity.

## Results

**Fig. 1:**
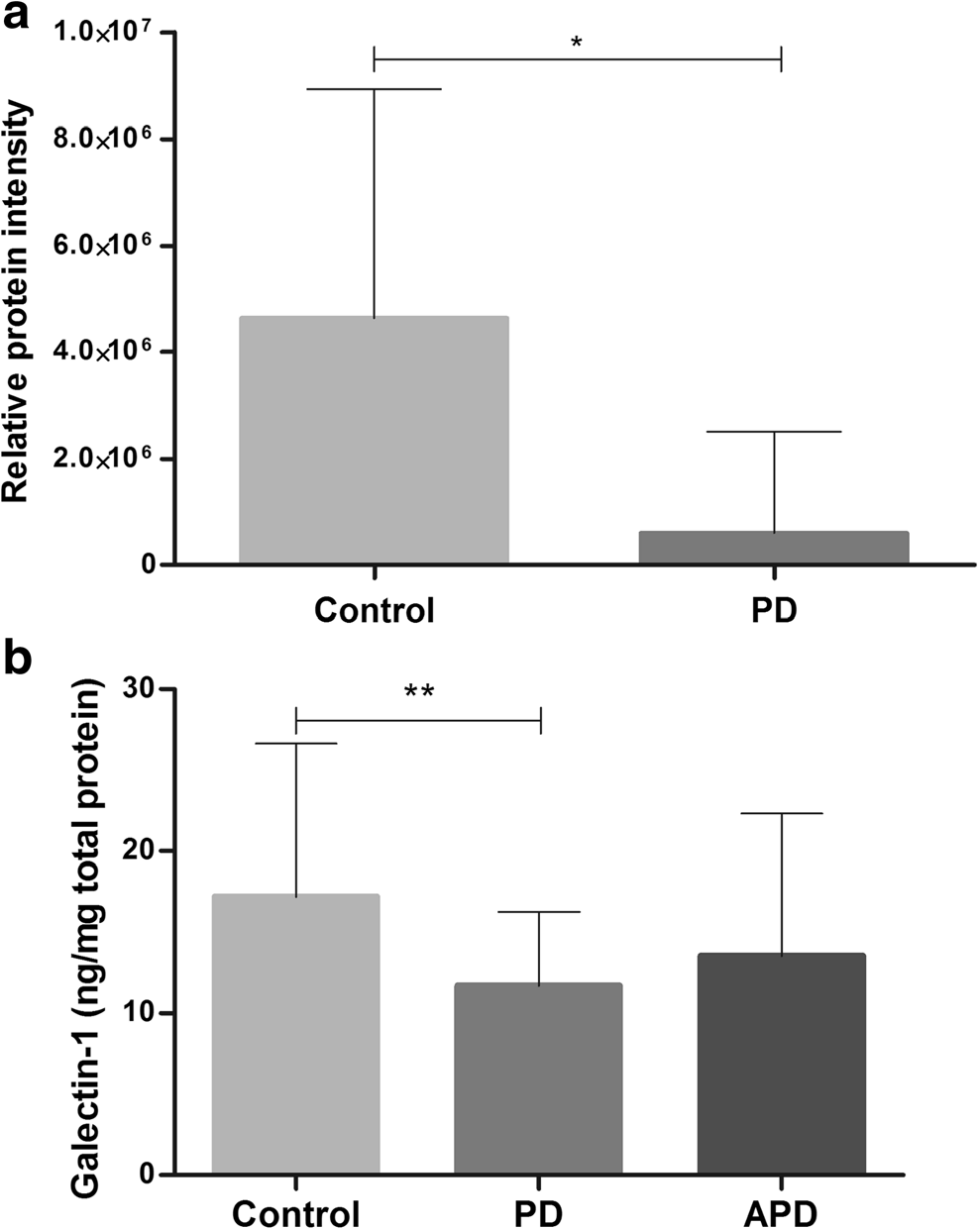
CSF Gal-1 levels in discovery (**a**) and validation (**b**) experiments for PD, APD, and non-neurological controls. **a** Relative protein intensity of Gal-1 found in profiling experiment by mass spectrometry showed decreased Gal-1 levels in PD compared to controls. **b** Gal-1 levels in CSF are lower in PD compared to controls in the validation study, but similar with APD. Gal-1 CSF levels were quantified by ELISA and normalized by total protein concentration. Data were analyzed using Mann–Whitney *U* test for discovery and Kruskal–Wallis test for validation; mean levels are shown with standard deviation, *p < 0.05; **p < 0.001

The aim of our study was to identify and validate a new biomarker for PD diagnosis. We first performed a profiling experiment by mass spectrometry in CSF of ten PD and ten non-neurological control patients. This discovery experiment resulted in the identification of 5543 peptides in CSF of both groups, corresponding to 872 different identified proteins. Only proteins quantified in at least five out of ten patients in any group (PD or control) were considered for further analysis, resulting in 482 proteins to be analyzed. Among these, 32 proteins were present at significantly different levels in PD and controls (*p* < 0.05) (Table S1). More details about the identification of peptides and proteins will be described in a separate study (manuscript in preparation). We selected Gal-1, which was present at significantly lower concentrations in PD (8×-fold lower), for further validation as a CSF biomarker candidate for the discrimination of PD and controls (*p* = 0.02) (Fig. 1a).

The performance of the ELISA assay was as follows. The detection limit was determined at 460 ng/L. The recovery of recombinant Gal-1 protein spiked in CSF was considered satisfactory with CV ranging from 85 to 118%. Both the intra-assay and inter-assay variation were considered satisfactory with a mean CV% of 4% (± 0.01) and 14% (± 0.05), respectively.

**Fig. 2:**
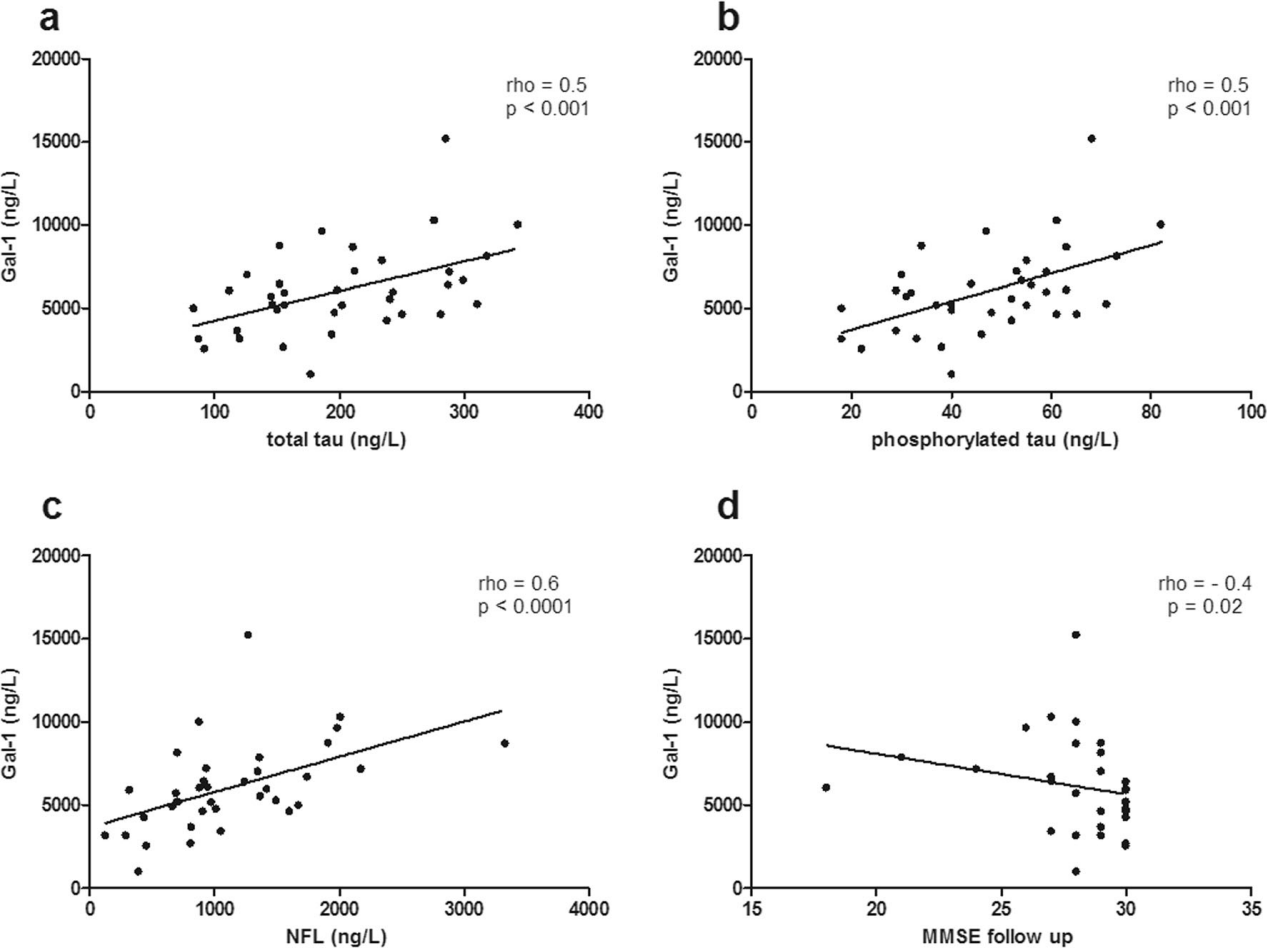
Correlation analysis in the PD group between Gal-1 and total tau, phosphorylated tau, NFL, and MMSE score. PD CSF Gal-1 levels were positively correlated to total tau (**a**), phosphorylated tau (**b**), and NFL (**c**). A negative correlation was found between Gal-1 and MMSE, a clinical parameter for cognitive impairment (**d**). Spearman’s rho coefficient value and *p* value for each correlation are indicated in the graphs

Age was significantly different between groups due to older patients in the APD group (*p* = 0.02). Total protein concentration was lower in PD and higher in APD patients than in controls (*p* < 0.001); therefore, Gal-1 levels were corrected for the total protein level. Gal-1 levels were lower in PD (mean 11 ng/mg total protein) compared to non-neurological controls (mean 17 ng/mg total protein, *p* < 0.003; Fig. 1b), but similar to APD (mean 14 ng/mg total protein). Gal-1 levels were similar in men and women in each group. Of note, Gal-1 levels in PD, APD, and controls were correlated with age (rho = 0.44, *p* < 0.0001). The difference in Gal-1 levels between the PD and controls remained significant after correction for age and intake of dopaminergic medication (*p* = 0.013). CSF Gal-1 levels in PD/APD patients were similar when they were either on dopaminergic medication or not at the time of lumbar puncture (*p* = 0.15). CSF Gal-1 levels in the PD group were positively correlated to CSF concentrations of total tau (rho = 0.53, *p* < 0.001), phosphorylated tau (rho = 0.54, *p* < 0.001), and NFL (rho = 0.58, *p* < 0.0001), but α-syn levels were not significantly correlated to Gal-1 (rho = − 0.11, *p* = 0.5). CSF Gal-1 levels correlated to MMSE scores determined at 3 years of follow-up (rho = − 0.44, *p* = 0.02; Fig. 2). Gal-1 CSF levels in the APD group were correlated only to disease duration (rho = 0.45, *p* = 0.04). No significant correlations between Gal-1 and other clinical parameters were observed. Partial analysis, taking age and total protein concentration as covariates, confirmed the correlations of Gal-1 in the PD group with total tau, phosphorylated tau, but not with NFL or MMSE. Correlation of Gal-1 levels to disease duration in the APD group was also retained.

## Discussion

We aimed to identify new CSF biomarkers for PD. We successfully performed a mass spectrometry profiling study using CSF of PD and controls, in which a total of 482 proteins were robustly identified. Among them, Gal-1 was considered as a potential biomarker for PD due to the strongly decreased levels in PD as compared to controls. We confirmed these differences by ELISA in an independent and larger validation cohort of PD, APD, and controls. Gal-1 levels in CSF had a moderate–high accuracy for discrimination of PD and controls. CSF Gal-1 levels were similar in PD and APD, however, indicating that Gal-1 levels in CSF may serve as a biomarker for parkinsonism, rather than for PD only.

Gal-1 is a member of the galectin family of proteins that binds to β-galactosides sugars. It is expressed in the nervous system during development, and a few other studies have reported expression of Gal-1 in the central and peripheral nervous system in adults [22–24]. Gal-1 expression in the nervous system has been correlated to proliferation of adult neural stem cells, astrocyte differentiation, and inflammation [25, 26].

In several reports, a neuroprotective role for Gal-1 has been described. Expression of Gal-1 was previously related to axonal and nerve cell regeneration after injury in animal models [23, 27, 28]. Administration of Gal-1 in an animal model of amyotrophic lateral sclerosis was associated with regeneration of spinal motor neurons, improvement of motor symptoms, and delay of disease onset [29].

The role of Gal-1 in PD or in APD is not yet clear, and only two studies reported findings in PD. One study showed reduction of Gal-1 levels in a cell model of PD, consisting of PC12 cells that were treated with a proteasomal inhibitor to promote ubiquitin–proteasome dysfunction, reproducing a general characteristic of PD [30]. Reduction of Gal-1 in this PD cell model may indicate the loss of neuroprotection after proteasomal inhibition. In another study, high Gal-1 levels were reported in PD substantia nigra [31]. These results are in apparent contrast with our study, since we observed decreased Gal-1 concentrations in the CSF of PD patients. The tissue study comprised five PD and five controls patients, with age ranging from 73 to 92 years in the PD group. No information about disease duration or other clinical parameters could be retrieved from this study, but at high age, PD is typically advanced to a severe stage. Thus, the contradictory outcomes of Gal-1 protein levels in brain tissue vs. CSF may be explained by the difference in sample type, a difference in disease stage (relatively early in our study vs. relatively late in the tissue study), and the younger population in our study which may affect Gal-1 concentrations in CSF. No reports have been published about a relation between Gal-1 protein levels in different brain regions as compared to CSF, or a relation between age and tissue levels of Gal-1.

The correlation of Gal-1 with either tau or NFL may indicate association with neuronal degeneration. Tau has previously been described as a potential biomarker for prediction of cognitive decline in PD, but this was not a consistent finding across multiple studies (reviewed in [32]). Overexpression of Gal-1 in mice with spinal lesions was correlated to an increase of tau levels and axonal regeneration of injured axons [33]. NFL has been widely studied as a biomarker for discrimination of PD from APDs with high accuracy levels (reviewed in [32]). Previous studies also indicated the involvement of NFL in axonal regeneration (reviewed in [34]). Based on literature, the correlation of Gal-1 with either tau or NFL, but not with α-syn levels in CSF, allows us to speculate a potential association of Gal-1 with axonal damage. However, future studies should be performed to investigate in more detail such as possible relation.

One limitation to our study may be the relatively small number of APD samples available for this study, which might have underestimated the power of Gal-1 to discriminate PD from APD. However, the design of our prospective study did not allow for an inclusion of a larger number of APD patients. Further confirmation in larger, independent, cohorts remains necessary.

Another limitation is that our CSF samples were stored for a long period since its collection, between 2003 and 2006, and long-term storage may affect protein stability. Protein levels are generally low in CSF, and differences in sample processing could affect the results of mass spectrometry and ELISA analysis. However, we followed international guidelines for CSF collection and storage that were described later in a consensus paper [35]. Previous studies reported increased protein instability in CSF samples storage at − 20 °C [35]; however, our samples were stored at − 80 °C. A recent study with Alzheimer’s disease CSF samples stored at − 80 °C for up to 12 years showed that CSF concentration of amyloid β, total, and phosphorylated tau proteins remained stable during this CSF storage time [36]. Therefore, we assume that the extended storage time has not influenced our results.

An important strong aspect of our study is that, although the number of parkinsonism patients we included in this study is moderate, they were selected from a unique longitudinal study. Unlike many other biomarker studies, which use highly selected cohorts of patients with clinically undisputable diagnosis, this study included patients with uncertain diagnosis at baseline, closely representing the daily situation for clinicians when they are confronted with a patient suspected of a movement disorder [7]. In these situations, biomarkers are mostly needed. Therefore, our cohort offers a great and relevant basis for biomarker discovery and validation.

In summary, we successfully profiled proteins present in CSF of PD and non-neurological controls by mass spectrometry. Among the proteins that were differentially expressed in PD vs. controls, Gal-1 was selected as a potential biomarker for PD. Our validation experiment for Gal-1 confirmed our findings from the discovery study, indicating that mass spectrometry profiling of proteins in CSF may be a useful tool to indentify novel biomarkers of neurological diseases, but this validation also demonstrated that CSF Gal-1 levels were similar in PD and APD. Furthermore, the correlations of Gal-1 with both NFL and tau suggested that Gal-1 may be involved in axonal function; however, further studies should clarify this association.

## Electronic Supplementary Material

ESM 1 (DOCX 18 kb)
