# Supplementary material for: Cerebrospinal Fluid Galectin-1 Levels Discriminate Patients with Parkinsonism from Controls
Source: Mol Neurobiol. 2018 Nov 21;56(7):5067–74. doi: 10.1007/s12035-018-1426-9 (PMC6647396; doi:10.1007/s12035-018-1426-9)
Supplement: Supplementary file 1 — (DOCX 18 kb) [file 12035_2018_1426_MOESM1_ESM.docx]

**Cerebrospinal fluid galectin-1 levels discriminate patients with Parkinsonism from controls**

**Molecular neurobiology**

Tainá M. Marques^1,2,3^; Anouke van Rumund^1,3^; Ilona B. Bruinsma^1,2^; Hans J.C.T. Wessels^2^; Jolein Gloerich^2^; Rianne A. J. Esselink^1,3^; Bastiaan R. Bloem^1,3^; H. Bea Kuiperij^1,2^; Marcel M. Verbeek^1,2,3^*

^1^Department of Neurology, Donders Institute for Brain, Cognition and Behaviour, Radboud University Medical Center, Nijmegen, The Netherlands

^2^Department of Laboratory Medicine, Radboud University Medical Center, Nijmegen, The Netherlands

^3^Parkinson Center Nijmegen, Nijmegen, The Netherlands

*Correspondence to: Dr. Marcel M. Verbeek, Department of Neurology, 830 TML, Radboud University Medical Center, P.O. Box 9101, 6500 HB Nijmegen, the Netherlands. Tel.: +31 243614567; Fax: +31 2436 68754; E-mail address: Marcel.Verbeek@radboudumc.nl; Website: [www.neurochemistry.nl](http://www.neurochemistry.nl)

Caption: List of the 32 proteins differently expressed between Parkinson's disease and controls

| **Table S1**. Proteins differently expressed between Parkinson's disease and controls | | |  |  |
| --- | --- | --- | --- | --- |
| **Proteins** | **Control** | **PD** | **p-value^a^** | **Ratio** |
| selenoprotein P | 2.4E+07 ± 5.4E+06 | 3.3E+07 ± 5.8E+06 | 0.002 | 1.4 |
| glyceraldehyde-3-phosphate dehydrogenase | 1.1E+07 ± 7.9E+06 | 2.1E+06 ± 3.6E+06 | 0.002 | 5.5 |
| laminin subunit beta-2 | 4.5E+06 ± 2.5E+06 | 7.3E+06 ± 2.8E+06 | 0.002 | 1.6 |
| calbindin | 2.9E+07 ± 1.1E+07 | 1.3E+07 ± 1.4E+07 | 0.007 | 2.3 |
| aspartate aminotransferase, mitochondrial | 4.4E+07 ± 2.4E+07 | 1.0E+07 ± 1.7E+07 | 0.007 | 4.2 |
| coagulation factor XIII B chain | 1.3E+07 ± 8.8E+06 | 3.5E+06 ± 4.7E+06 | 0.009 | 3.6 |
| calmodulin-like protein 5 | 1.0E+05 ± 2.7E+05 | 3.3E+06 ± 5.7E+06 | 0.010 | 32.4 |
| V-set and immunoglobulin domain-containing protein 4 | 3.2E+07 ± 1.6E+07 | 1.1E+07 ± 1.5E+07 | 0.013 | 2.8 |
| mimecan | 4.6E+08 ± 1.1E+08 | 6.3E+08 ± 1.6E+08 | 0.016 | 1.4 |
| N(4)-(beta-N-acetylglucosaminyl)-L-asparaginase | 1.1E+07 ± 1.1E+07 | 1.3E+06 ± 4.1E+06 | 0.017 | 8.9 |
| galectin-1 | 4.6E+06 ± 4.3E+06 | 6.0E+05 ± 1.9E+06 | 0.017 | 7.7 |
| fibulin-5 | 4.2E+07 ± 2.4E+07 | 8.2E+07 ± 5.4E+07 | 0.021 | 1.9 |
| glutathione synthetase | 5.5E+06 ± 2.9E+06 | 2.2E+06 ± 3.1E+06 | 0.025 | 2.6 |
| complement factor D | 1.2E+07 ± 1.1E+07 | 2.9E+06 ± 4.8E+06 | 0.026 | 4.2 |
| semaphorin-6D | 2.9E+06 ± 2.2E+06 | 9.7E+05 ± 1.3E+06 | 0.029 | 3.0 |
| 14-3-3 protein zeta/delta | 4.7E+06 ± 4.5E+06 | 7.6E+06 ± 3.0E+06 | 0.031 | 1.6 |
| hepatitis A virus cellular receptor 2 | 5.3E+06 ± 3.8E+06 | 1.8E+06 ± 3.2E+06 | 0.032 | 2.9 |
| cocaine- and amphetamine-regulated transcript protein | 9.6E+06 ± 7.6E+06 | 2.3E+07 ± 1.6E+07 | 0.033 | 2.4 |
| coagulation factor IX | 1.9E+07 ± 1.4E+07 | 8.6E+06 ± 1.1E+07 | 0.034 | 2.2 |
| fetuin-B | 1.0E+07 ± 7.3E+06 | 3.9E+06 ± 8.1E+06 | 0.035 | 2.6 |
| carbohydrate sulfotransferase 15 | 7.8E+06 ± 5.9E+06 | 2.7E+06 ± 4.4E+06 | 0.039 | 2.9 |
| proenkephalin-A | 1.1E+07 ± 7.1E+06 | 1.7E+07 ± 5.5E+06 | 0.041 | 1.6 |
| complement C1r subcomponent | 2.2E+08 ± 6.4E+07 | 1.7E+08 ± 2.7E+07 | 0.041 | 1.3 |
| plexin-B2 | 1.4E+07 ± 3.2E+06 | 1.8E+07 ± 5.4E+06 | 0.041 | 1.3 |
| netrin-G1 | 1.9E+07 ± 1.3E+07 | 7.5E+06 ± 1.2E+07 | 0.045 | 2.5 |
| fibrinogen gamma chain isoform gamma-A | 3.7E+06 ± 2.9E+06 | 1.8E+06 ± 3.2E+06 | 0.045 | 2.1 |
| kunitz-type protease inhibitor 1 | 3.7E+06 ± 2.7E+06 | 1.1E+06 ± 2.3E+06 | 0.047 | 3.4 |
| 14-3-3 protein epsilon | 1.4E+07 ± 7.7E+06 | 6.8E+06 ± 8.2E+06 | 0.048 | 2.1 |
| cathepsin L1 | 3.0E+07 ± 1.6E+07 | 1.8E+07 ± 1.2E+07 | 0.049 | 1.7 |
| neogenin isoform 2 | 9.5E+07 ± 2.0E+07 | 7.2E+07 ± 1.8E+07 | 0.049 | 1.3 |
| leucine-rich repeat-containing protein 4B | 1.3E+08 ± 2.0E+07 | 1.0E+08 ± 3.2E+07 | 0.049 | 1.3 |
| lymphatic vessel endothelial hyaluronic acid receptor 1 | 1.0E+07 ± 9.8E+06 | 2.6E+06 ± 5.5E+06 | 0.049 | 4.0 |

Values are expressed by mean of arbitrary intensity ± standard deviation

PD: Parkinson's disease

^a^group differences were accessed by Mann-Whitney
